# Bladder rupture due to urogenital tract trauma caused by ox horn injury in a patient with pelvic organ prolapse: a case report

**DOI:** 10.1186/s13256-020-02552-0

**Published:** 2020-11-24

**Authors:** Zelalem Mengistu, Mezigebu Molla

**Affiliations:** Department of Obstetrics and Gynecology, University of Gondar, Gondar, Ethiopia

**Keywords:** Horn injury, Urogenital injury, Genital trauma, Bladder rupture, Pelvic organ prolapse injury

## Abstract

**Introduction:**

Genitourinary tract trauma caused by ox horn injury in the presence of pelvic organ prolapse (POP) is an extremely rare phenomenon and associated with devastating morbidity.

**Case presentation:**

A 50-year-old multiparous postmenopausal woman from rural northwest Ethiopia presented with the primary complaint of urinary incontinence 6 days after she suffered ox horn injury to her prolapsed genitalia. She had stage 3 pelvic organ prolapse with the leading point being the cervix. The anterior vaginal and posterior bladder walls were disrupted with visible draining of the left ureter. The wound was dirty and edematous with whitish discharge. She was admitted to the urogynecology ward and provided with wound care until the infection subsided. Apical prolapse suspension was performed using right sacrospinous fixation, and bladder repair was carried out 6 weeks following the prolapse suspension. She recovered well and was continent when discharged.

**Conclusion:**

Ox horn injury involving the female lower urogenital tract in the presence of POP is extremely rare. Late presentation after sustaining injury is associated with increased risk of morbidity and long hospital stay, and treatment requires multistage surgery.

## Introduction

Traumatic injury to the female genital tract includes external injuries to the labia, vulva or vagina, urethra and anus and internal injuries to the bony pelvis, bladder, bowels and reproductive organs [1]. Obstetric complications are the most common cause of female urogenital trauma, although non-obstetric causes are not unusual. However, non-obstetric injury to the lower genitourinary tract in females has not been well described in the literature. The non-obstetrics causes of genital trauma are classified as coital or non-coital. The majority of non-coital causes result from striking injuries (edge of chairs, stools, sharp object etc.), road traffic accidents, violence, bull horn injury and genital mutilation [2, 3]. The risk factors associated with non-birth-related injury to the genital tract are age, marital status, residential location, occupation, socioeconomic status, leisure and sporting activities and sexual behavior [4].

Although physical traumas resulting from cattle horn injuries are relatively common on various parts of the body, injuries to the genitalia and lower urinary system from cattle horns are rarely reported [5, 6].

Urogenital tract trauma accounts for 10% of all abdominopelvic traumatic injuries of which bladder injury occurs in 1.6% of these cases [7, 8]. Injury to the bladder is not common due to the protection provided by the bony pelvis. Bladder injury is usually associated with a high-impact trauma [8, 9]. Among bladder injuries, bladder rupture is a rare phenomenon. Bladder rupture can be either extraperitoneal (EP) or intraperitoneal (IP). EP ruptures are more common and usually result from forceful impact to the anterior bladder [8, 9], while IP ruptures usually result from a rise in intravascular pressure following an abdominopelvic impact that causes rupture of one of the weaker points of the bladder, such as the dome [10].

We present a case of bladder rupture in a postmenopausal woman with stage three pelvic organ prolapse (POP) that resulted from an injury caused by an ox horn. The patient was successfully treated and was continent at discharge.

Written informed consent was obtained from the patient for publication of this case report and accompanying images.

## Case presentation

A 50-year-old postmenopausal woman, gravida 15, para 7, from a rural area in northwest Ethiopia presented to the University of Gondar Urogynecology unit after she sustained an ox horn injury to her genitalia 6 days prior to presentation. She initially had profuse bleeding from the injury site, but the bleeding stopped spontaneously and she did not seek medical care until she presented to our urogynecology unit. At presentation, she reported a history of failure to control urine and pain, and a foul-smelling discharge at the wound site. She had history of a mass protruding outside the vagina of 7 years duration that was progressively increasing and associated with difficulty in urinating for which she had not sought medical care. Following the injury she denied abdominal pain, abdominal distension or failure to pass feces and flatus. Her last menses was 2 years prior to the injury. She had no known history of other medical problems or illness.

**Fig. 1:**
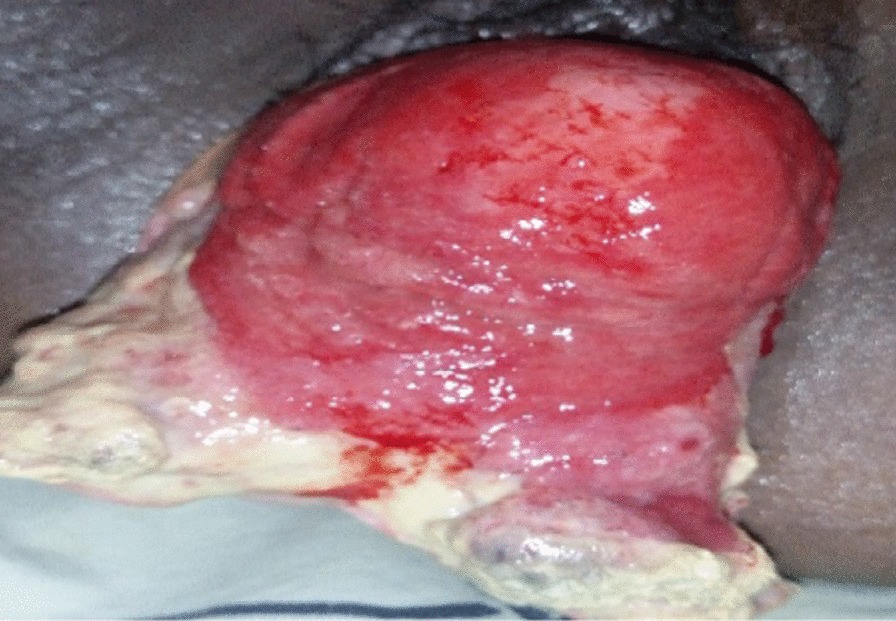
Edematous and infected ruptured bladder at admission

**Fig. 2:**
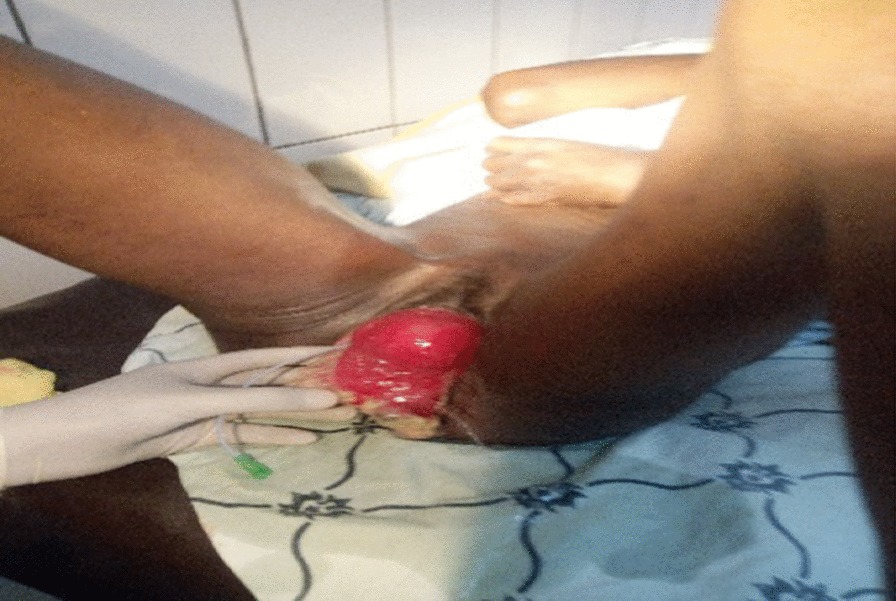
Stented right ureter

On physical examination she was acutely sick looking, vital signs were within normal range and she had slightly pale conjunctiva. Her abdomen was flat, non-tender and with active bowel sounds with no palpable mass or sign of fluid collection. Evaluation of the genitourinary system revealed stage three POP with the leading point being the cervix. The anterior vaginal and posterior bladder walls were disrupted with visible draining of the left ureter. The wound was dirty and edematous with a whitish discharge (Fig. 1). There was no involvement of the urethra and the distal one-third of the vagina in the injury. She had difficulty walking due to the pain from the wound. Otherwise there were no remarkable findings from her physical examination. Her hematocrit was 31.6%, and abdominopelvic ultrasound revealed a mild right-sided hydronephrosis with hydroureter. There was no sign of intra-abdominal or pelvic fluid collection. She was admitted to the urogynecology ward with a diagnosis of stage three POP, posterior bladder rupture and wound infection. The right ureteral orifice was identified and stented (Fig. 2), and she received wound care twice daily.

**Fig. 3:**
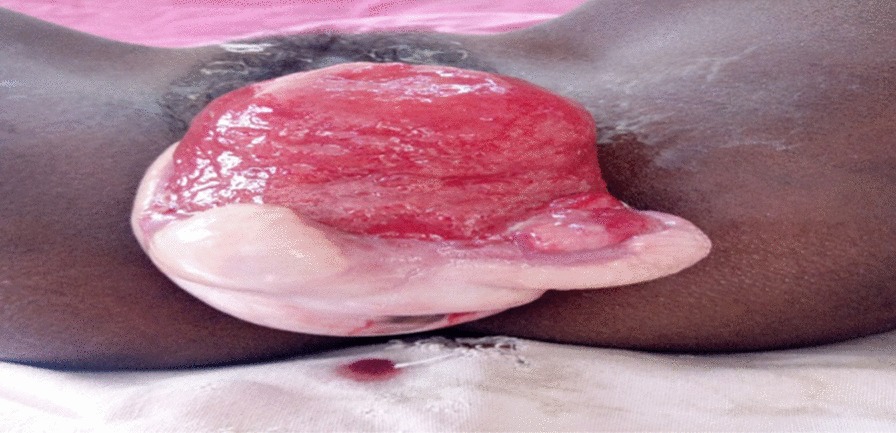
Stage III pelvic organ prolapse with ruptured bladder before right sacrospinous ligament fixation

**Fig. 4:**
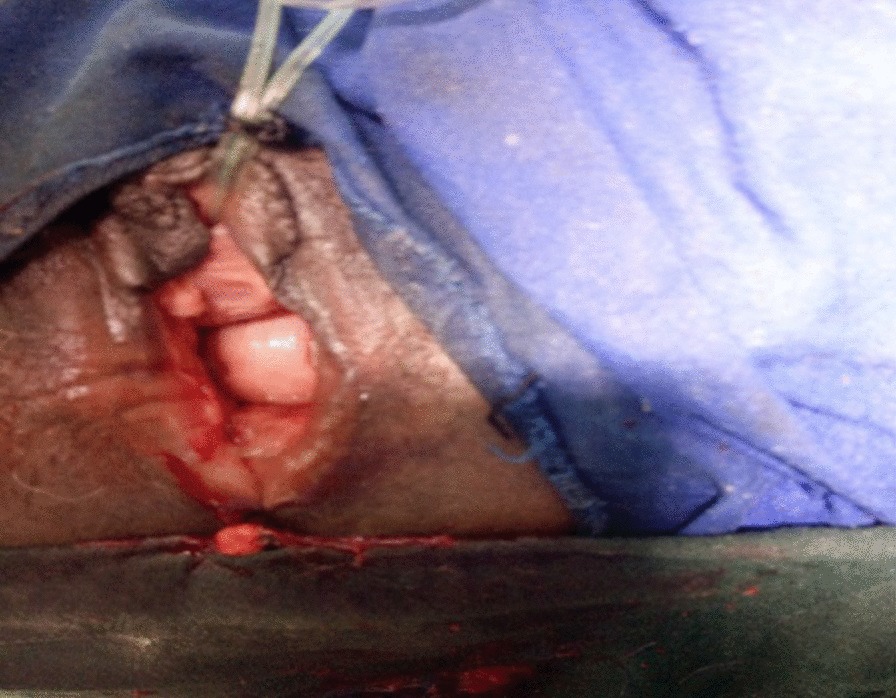
After apical repair with right sacrospinous ligament fixation

Three months following admission, the wound was clean and well granulated and the edema had resolved. The right sacrospinous ligament fixation was then completed (Figs. 3 and 4).

**Fig. 5:**
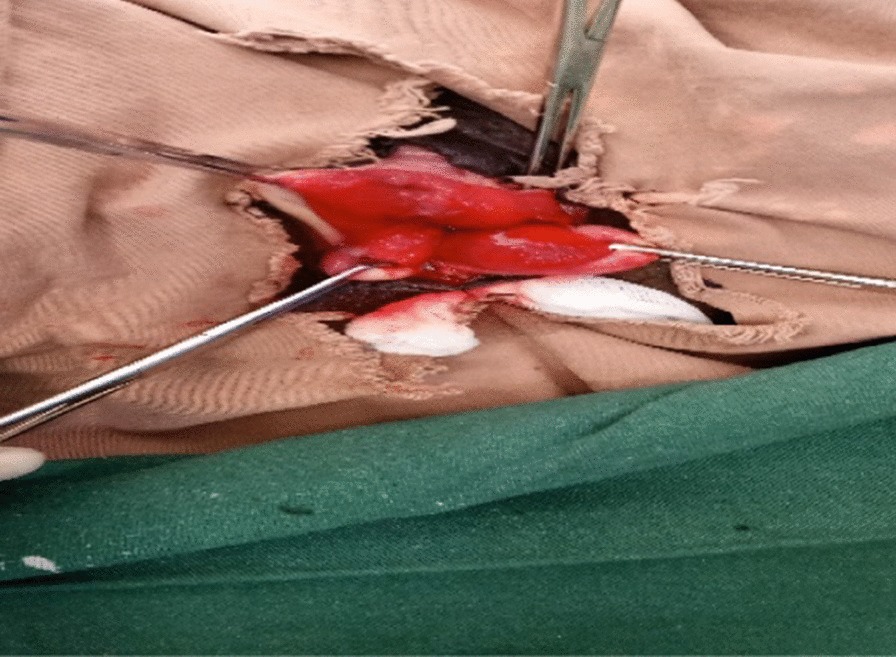
Ruptured bladder before repair

Six weeks after the RSSF the ruptured bladder was repaired after adequate mobilization of the bladder (Fig. 5). Both the ureters were identified and stented prior to the repair. Vaginal packing and transurethral catheter were left in situ. The vaginal packing and the urinary catheter were removed after 24 hours and 3 weeks, respectively. The patient was fully continent when discharged on postoperative day 21 following bladder repair and on postoperative day 63 following her RSSF.

## Discussion

Physical trauma to the urogenital system due to horn injury is a rare phenomenon as the level of this system is lower than the head of the bull and the perineum is a highly protected region due to the reflex adduction of the thigh [5, 11]. However, depending on the relative positions of the bull’s head and the victim an injury may occur to any part of the body [12]. This is clearly illustrated in our case, in which the patient was in squatting position preparing food for her cattle. A 5-year retrospective review of hospital data on lower genitourinary tract trauma in females caused by cow horn injury identified only 12 cases of such injuries, and none involved the bladder [6].

The relevance of this case is its uniqueness as no similar case has been reported to date. Lacerations to the female lower genitourinary system caused by bull horn injuries are usually limited to the lower vagina because the horns are long, curved and directed forwards with tapering edges. Bull horns can also penetrate body cavities, such as the bladder [4, 5, 13]. Among possible bladder injuries, bladder rupture resulting from horn injury, as seen in our patient, is an extremely rare phenomenon. In the case of our patient, however, the rupture of the bladder was due to the penetrative horn injury disrupting the anterior vaginal wall and the base of the bladder. One possible contributory factor to this atypical injury may have been the presence of advanced stage POP in this woman. The slow healing of the wound was due to the late presentation and the postmenopausal state. She stayed at the hospital for > 3 months due to two-staged surgery because postmenopausal hypo-estrogenic changes had induced thinning and atrophy of the vulvo-vaginal epithelium as well as vaginal lubrication [4].

## Conclusion

Ox horn injury involving the female genitourinary tract and resulting bladder rupture is extremely rare. The presence of advanced-stage POP increases the risk of severe complications. Late presentation will result in delayed repair and a longer hospital stay.

## Abbreviations

EP: Extraperitoneal
IP: Intraperitoneal
POP: Pelvic organ prolapse
RSLF: Right sacrospinous ligament fixation
